# Dual Inhibition of PI3 Kinase and MAP Kinase Signaling Pathways in Intrahepatic Cholangiocellular Carcinoma Cell Lines Leads to Proliferation Arrest but Not Apoptosis

**DOI:** 10.3390/cimb46070439

**Published:** 2024-07-13

**Authors:** Jessica Schüler, Martina Vockerodt, Niloofar Salehzadeh, Jürgen Becker, Jörg Wilting

**Affiliations:** Institute of Anatomy and Embryology, University Medical Center Goettingen, GAU, 37075 Goettingen, Germany; jessica.schueler@stud.uni-goettingen.de (J.S.); martina.vockerodt@med.uni-goettingen.de (M.V.); niloofar.salehzadeh@med.uni-duesseldorf.de (N.S.); juergen.becker@med.uni-goettingen.de (J.B.)

**Keywords:** cholangiocyte, primary liver cancer, selumetinib, AZD6244, MK2206, intrahepatic cholangiocarcinoma

## Abstract

Cholangiocellular carcinoma (CCA) is the second most common primary liver cancer, with increasing incidence worldwide and inadequate therapeutic options. Intra- and extrahepatic bile ducts have distinctly different embryonic origins and developmental behavior, and accordingly, intra- and extrahepatic CCAs (ICC vs. ECC) are molecularly different. A promising strategy in oncotherapy is targeted therapy, targeting proteins that regulate cell survival and proliferation, such as the MAPK/ERK and PI3K/AKT/mTOR signaling pathways. Inhibitors of these pathways have been tested previously in CCA cell lines. However, these cell lines could not be clearly assigned to ICC or ECC, and the results indicated apoptosis induction by targeted therapeutics. We tested targeted therapeutics (selumetinib, MK2206) in three defined ICC cell lines (HuH28, RBE, SSP25). We observed additive effects of the dual inhibition of the two pathways, in accordance with the inhibition of phospho-AKT and phospho-ERK1/2 expression. Proliferation was blocked more effectively with dual inhibition than with each single inhibition, but cell numbers did not drop below baseline. Accordingly, we observed G1 phase arrest but not apoptosis or cell death (measured by cleaved caspase-3, AIFM1 regulation, sub-G0/G1 phase). We conclude that the dual inhibition of the MAPK/ERK and PI3K/AKT/mTOR pathways is highly effective to block the proliferation of ICC cell lines in vitro; however, potential clinical applications must be critically examined, as a proliferation block could also induce resistance to standard therapies.

## 1. Introduction

Cholangiocellular carcinoma (CCA) is a tumor arising from cholangiocytes in the bile ducts. It accounts for 3–5% of gastrointestinal cancers and is the second most common liver tumor after hepatocellular carcinoma (HCC) [1,2,3]. In CCA, a distinction must be made between intrahepatic (ICC) and extrahepatic (ECC) tumors depending on their anatomical localization [4]. ICC arises from bile ducts deeply within the liver, whereas ECC involves the larger perihilar and extrahepatic (distal) bile ducts [5,6]. Thereby, perihilar CCA is the most common form, accounting for 50–60% of CCAs, while 20–30% are distal CCAs. ICC accounts for 10% of all primary liver tumors [1]. Even though ICC remains a rare tumor, its incidence has been increasing worldwide in the last 30 years, e.g., in the USA, by approximately 165% to currently 0.95 per 100,000 inhabitants [7]. The majority of CCAs, approximately 70%, develop for no apparent reason. However, there are a number of risk factors such as liver flukes *Opisthorchis viverrini* and *Clonorchis sinensis*, whose cercariae are transmitted through eating raw freshwater fish. Liver flukes occur primarily in East Asia and increase the risk of contracting CCA [4,8]. Primary sclerosing cholangitis (PSC) and hepatitis B and C with associated cirrhosis, diabetes, or obesity are other risk factors [9]. Strikingly, in industrial countries, ICC often occurs in the absence of cirrhosis [5]. The diagnosis of ICC is difficult because symptoms appear late and tend to be nonspecific, such as weight loss, abdominal discomfort, jaundice, malaise, or hepatomegaly. A curative treatment option for ICC is surgical resection. However, this is only possible in early stages and can therefore be performed in no more than 20–40% of cases. In the majority of patients, the tumor is too advanced for resection [10]. The first-line chemotherapy for unresectable CCA consists of gemcitabine and cisplatin, and second-line chemotherapeutics can be effective in selected patients [11].

A promising strategy in cancer therapy is targeted therapy, which targets proteins that regulate cell survival and proliferation [12,13]. Although this concept has been proposed for CCA quite some time ago [14,15], it has not yet been firmly established in clinics. In particular, the molecular heterogeneity of ECC and ICC must be taken into account [16], which is very likely due to the fact that extra- and intrahepatic bile ducts develop differently in the embryo. The embryonic development, differentiation and migration potency of the respective cell has a major influence on the pathophysiology. While the extrahepatic bile duct emerges directly by sprouting from the foregut endoderm, the intrahepatic bile ducts develop from bipotent progenitor cells, which transiently form a sheath of epithelial cells called the ductal plate. Only later do the cells acquire a tubular morphology [17,18]. Therefore, ICC and ECC must be regarded as heterogenous tumor entities. For targeted therapy, this must be taken into account. For ICC patients, targeted therapy (with pemigatinib) has only been approved for a small cohort of patients with gene fusions affecting *fibroblast growth factor receptor 2* (*FGFR2*) [19].

A frequently used strategy is the targeted inhibition of the MAPK/ERK signaling pathway, which is hyperactive in at least 40% of cancers [20]. Furthermore, the PI3K/AKT/mTOR signaling pathway is highly active in numerous types of cancer [21]. Although the effects of the inhibition of the two pathways were tested in CCA in vitro [15], the three human cell lines used in this study were either ECC (one cell line) or cannot be clearly assigned to ECC or ICC. Here, we focused on ICC and the cell lines HuH28, RBE and SSP25. The three cell lines represent the spectrum of spindle-shaped to epithelial-like cell morphology typically found in ICC (for references, see below in Materials and Methods). We studied the AKT inhibitor MK2206 and the MEK inhibitor selumetinib and determined their single and dual effects on the cell cycle, proliferation, the phosphorylation of signaling molecules and apoptosis. We observed additive effects of dual pathway inhibition on proliferation in conjunction with a G1 phase arrest but no apoptosis. Therefore, cell counts never dropped below baseline. In sum, dual pathway inhibition is highly effective in blocking the proliferation of ICC in vitro, but it must obviously be performed in conjunction with a standard therapy that kills the remaining tumor cells.

## 2. Materials and Methods

### 2.1. Cell Culture

The human ICC cell lines (HuH28, RBE, SSP25) were purchased freshly from RIKEN BioResources Research Center (Tsukuba, Japan). The three cell lines represent the spectrum of spindle-shaped to epithelial-like cell morphology typically found in ICC. HuH28 is an intrahepatic CCA line with mainly spindle-cell morphology and a small percentage of polygonal-shaped cells, maintained at RIKEN since 1995 [22,23]. SSP-25 is an intrahepatic CCA line, with fibroblast-like morphology, maintained at RIKEN since 1996 and used in multiple studies [24]. RBE is an intrahepatic CCA line with epithelial-like morphology, maintained at RIKEN since 1996 and used in multiple studies [25,26]. The cells were incubated at 37 °C with 5% CO_2_ under humidified atmosphere in RPMI medium (10% FCS, 1% penicillin/streptomycin). Cells were amplified and used usually during passages 9–10 but not beyond passage 16.

### 2.2. Proliferation Assay and Drug Preparation

Proliferation assays were performed in 96-well plates as described previously [27]. Cells were seeded at a concentration of 5 × 10^3^ cells/mL in 100 µL RPMI and incubated at 37 °C overnight. Cells were treated with the inhibitors MK2206 and selumetinib (AZD-6244) (both from Selleckchem, Munich, Germany) and DMSO (control). For single treatment, 0.1 µM, 0.5 µM, 1 µM and 5 µM were used. Dual treatment: 0.5 µM (each) MK2206+selumetinib and 1 µM (each) MK2206+selumetinib were used. The stock solution was 10 mM of the respective drug dissolved in DMSO. Stock solutions were then diluted with cell medium to the respective final concentration. DMSO controls contained the same amount of DMSO as in the highest inhibitor concentration.

After 0 h, 24 h, 48 h and 72 h, cells were fixed with 5% glutaraldehyde and stained with crystal violet. After drying, 10% acetic acid was added and incubated for 15 min. Extinction was measured at 595 nm with an iMark™ Microplate Absorbance Reader (Bio-Rad, Tokyo, Japan). Extinction was measured at various time points, normalized to the extinction at 0 h, and converted to percent. Experiments were repeated at least 3 times with 8 replicates each.

### 2.3. Protein Extraction and Western Blot

Cells were seeded at concentrations of 3 × 10^6^ cells/mL in RPMI in cell culture dishes and incubated for 12 h at 37 °C. Then, cells were treated with inhibitors (1 µM) for 12 h and washed with PBS containing 1 mM sodium orthovanadate on ice. Lysis was performed with a lysis buffer mixture containing RIPA lysis buffer (aqua dest., 140 mM NaCl, 10 mM TrisHcl pH 8, 1 mM EDTA, 1% Triton, 0,1% SDS, 0,1% Sodiumdeox) with 1 mM SOV and 1× Sample Complete Protease Inhibitor (Roche Diagnostics) for 30 min on ice. Lysates were centrifuged at 14,000 rpm for 15 min at 4 °C, and the supernatant was transferred. Protein concentration was measured by the Pierce BCA protein assay according to the manufacturer’s instructions (Thermo Fisher Scientific, Waltham, MA, USA). Sodium dodecyl sulfate polyacrylamide gel electrophoresis (SDS-Page) was performed. A total of 20 µg of protein was denatured at 70 °C for 10 min. Proteins were transferred onto a PVDF membrane and incubated with a primary antibody (Table 1) overnight at 4 °C followed by washing processes. Then, secondary horseradish peroxidase (HRP)-conjugated antibodies were incubated for 1 h at RT. Visualization was performed with an enhanced chemiluminescence (ECL) solution (SignalFire™ ECL Reagent or Clarity Western ECL Substrate) in BioRad ChemiDoc (Bio-Rad). WB analyses were repeated at least three times, and luminescence signals were quantified using Image Lab Software 6.0.1 (Bio-Rad).

### 2.4. Semiquantitative Real-Time PCR (qPCR)

Cells were treated for 24 h with 1 µM MK2206 or selumetinib or with 1 µM of each drug.

For RNA isolation, the medium was removed, and cells were washed with PBS. NucleoZOL (Machery-Nagel) was used according to the manufacturer’s instructions. A total of 2 µg of RNA was transcribed with Qiagen Omniscript reverse transcriptase (QIAGEN). The relative RNA expression was determined with the 2^−ΔΔCT^ method [28]. Experiments were performed three times in duplicate. The following primers were used to detect Apoptosis-inducing factor mitochondria-associated 1 (AIFM1); Fwd: 5′-TGGGCTTATCAACAGTAGGAGC-3′; Rev: 5′-TTCTGGTGTCAGCCCTAACC-3′, Actin Fwd: 5′-GCATCCCCCAAAGTTCACAA-3′; Rev: 5′-AGGACTGGGCCATTCTCCTT-3′.

### 2.5. Flow Cytometry

Cells were seeded in 6-well plates in concentrations of 8 × 10^4^ cells/mL and were treated with inhibitors or DMSO (control) after 24 h and were further incubated for 24 h. Both adherent cells and centrifuged supernatant containing floating (dying) cells were washed with PBS and suspended in Nicoletti buffer with 50 µg/mL propidium iodide (PI). Flow cytometry was measured with the BD LSRFortessa™ X-20. The maximum number of detectable events was 5000. Experiments were repeated 5 times. Cell cycle analysis was performed using FlowJo^TM^ Software V10.

### 2.6. Statistical Analysis

The data were analyzed with Graph Prism 5 software and Microsoft Excel 2019 MSO 16.78.3. The standard deviation (SD) was calculated for all experiments. Statistical significance was calculated with two-way ANOVA (proliferation assays) or one-way ANOVA (qPCR, flow cytometry) between the treatment and DMSO control groups. The statistical level of significance is shown as follows: * *p* ≤ 0.05, ** *p* ≤ 0.01, *** *p* ≤ 0.001, with a 95% level of confidence.

## 3. Results

### 3.1. AKT Inhibitor MK2206 Effectively Reduces Proliferation in ICC Cell Lines

**Figure 1:**
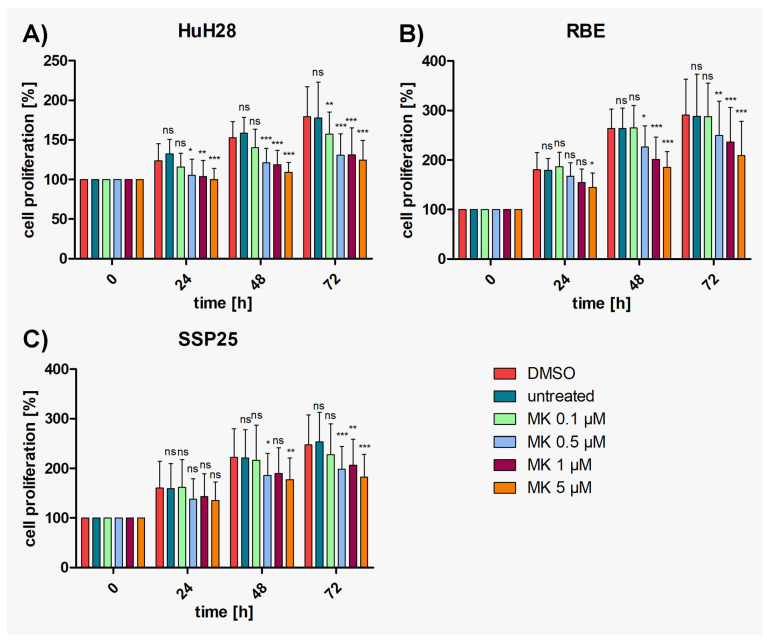
Proliferation assays of ICC cell lines treated with MK2206. (**A**) HuH28; (**B**) RBE; (**C**) SSP25. Cell numbers were calculated after 0, 24, 48 and 72 h with different concentrations of MK2206. Medium and DMSO controls are shown. Statistical analyses were performed compared to DMSO controls. Mean of *n* = 3 independent experiments with eight replicates are shown, as well as standard deviation and statistical significance. Note reduction in proliferation, but no drop below initial cell numbers. ns = not significant. * *p* ≤ 0.05, ** *p* ≤ 0.01, *** *p* ≤ 0.001.

To determine the effects of MK2206, proliferation assays were performed with concentrations ranging from 0.1 µM to 5 µM, in addition to previous studies on 10 µM and 25 µM [29]. The results show that the numbers of all three ICC cell lines (HuH28, RBE, SSP25) increased steadily over time in both the medium control group and the DMSO control group. The IC50 (95% CI) values after 72 h treatment were similar for all three cell lines: HuH28 5.92 µM (3.37–10.41); RBE 6.09 µM (2.9–12.69) and SSP25 5.08 µM (2.8–9.2). MK2206 application induced a dose-dependent inhibition of cell proliferation in all cell lines (Figure 1). Highly significant inhibition in comparison with the DMSO control was observed at 0.5 µM after 24 h in HUH28 cells, resulting in 15% growth inhibition (Figure 1A and Supplementary Figure S1A). Cell counts after inhibition with 0.5 µM–5 µM MK2206 reached the baseline (100%) but did not drop below this value (Figure 1A). MK2206 induced highly significant inhibition at 1 µM after 24 h in RBE cells as well, again with 15% growth inhibition (Figure 1B and Supplementary Figure S1B). In SSP25, significant effects were noticed at 0.5 µM after 48 h (Figure 1C and Supplementary Figure S1C). In sum, all three cell lines responded very well to treatment with MK2206, with slight differences in the dose and treatment time.

### 3.2. MEK Inhibitor Selumetinib Significantly Reduces Proliferation of HuH28 and RBE but Less in SSP25

**Figure 2:**
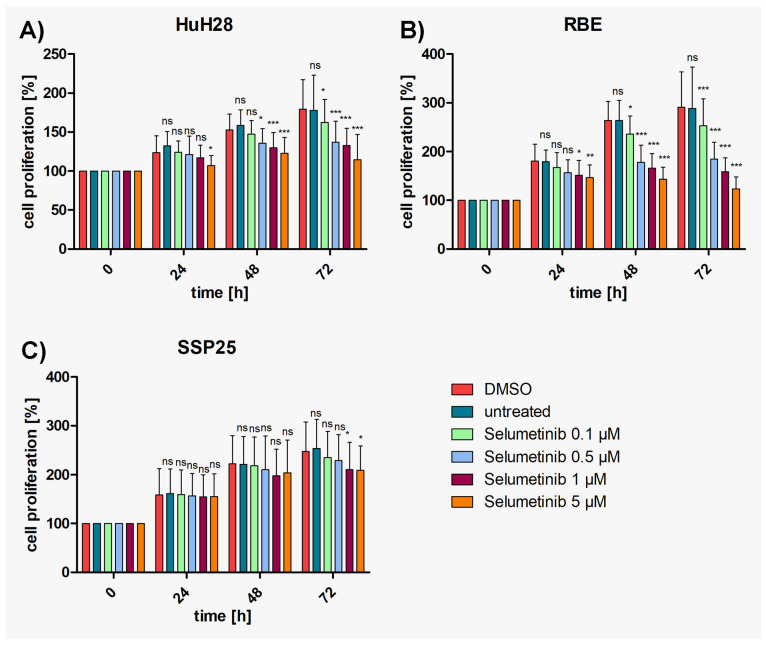
Proliferation assays of ICC cell lines with selumetinib. (**A**) HuH28; (**B**) RBE; (**C**) SSP25. Cell numbers were calculated after 0, 24, 48 and 72 h with different concentrations of selumetinib. Medium and DMSO controls are shown. Statistical analyses were performed compared to DMSO controls. Mean of *n* = 3 independent experiments with eight replicates are shown, as well as standard deviation and statistical significance. Note reduction in proliferation, but no drop below initial cell numbers. In SSP25, significant effects were seen only after 72 h. ns = not significant. * *p* ≤ 0.05, ** *p* ≤ 0.01, *** *p* ≤ 0.001.

In the controls, again, ICC cell lines (HuH28, RBE, SSP25) showed a steady increase in cell numbers over time. A dose-dependent inhibition of proliferation by selumetinib (AZD-6244) was observed in all three ICC cell lines, with HuH28 and RBE being more sensitive than SSP25. Again, cell numbers never dropped below baseline (Figure 2). Highly effective inhibition was observed in RBE cells with the highest significance (*p* ≤ 0.001) at 0.5 µM selumetinib after 48 h (Figure 2B), with 32% growth inhibition (Supplementary Figure S1B). In HuH28 cells, selumetinib inhibited cell proliferation highly significantly at 1 µM after 48 h, with 15% growth inhibition (Figure 2A and Supplementary Figure S1A). The effects of selumetinib were the weakest in SSP25. However, significant effects were observed at 1 µM after 72 h, with 15% growth inhibition (Figure 1C and Supplementary Figure S1C). In sum, HuH28 and RBE responded very well to treatment with selumetinib. SSP25 showed the highest resistance but responded after prolonged treatment.

### 3.3. Dual Inhibition Shows Additive Effects as Compared with Single Treatments

To investigate whether additive or synergistic effects could be achieved, concentrations of either 0.5 µM or 1 µM of MK2206 and selumetinib were tested in combination. In single treatment, these dosages induce between 10% and 35% inhibition (see Supplementary Figure S1). Highly significant inhibition in comparison with DMSO controls was achieved with both concentrations already after 24 h in HuH28 and RBE, which is earlier than with each single application (Supplementary Figure S2). Cell numbers nearly reached baseline values but barely dropped below. In SSP25, the combined application of 0.5 µM of inhibitors achieved a significant cell number reduction only after 72 h. With each 1 µM of MK2206 and selumetinib, robust effects were seen after 48 h (Supplementary Figure S2).

**Figure 3:**
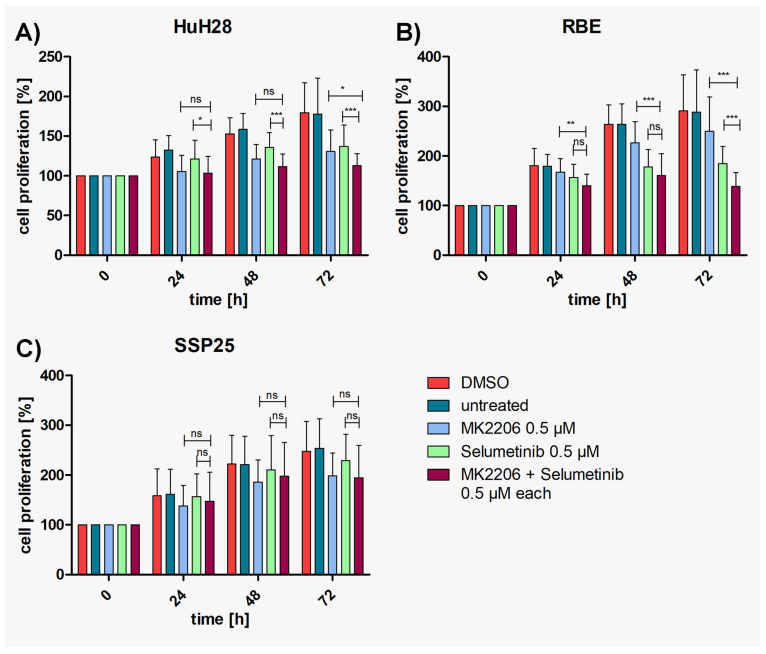
Proliferation assays of ICC cell lines with MK2206 and selumetinib, dual compared with single treatments. (**A**) HuH28; (**B**) RBE; (**C**) SSP25. Cell numbers were calculated after 0, 24, 48 and 72 h. Application of each 0.5 µM MK2206 and selumetinib. Mean of *n* = 3 independent experiments with eight replicates are shown, as well as standard deviation and statistical significance calculated with two-way ANOVA. Note highly significant effects of dual treatment in RBE after 72 h, significant effects in HuH28, but no effects in SSP25. ns = not significant. * *p* ≤ 0.05, ** *p* ≤ 0.01, *** *p* ≤ 0.001.

**Figure 4:**
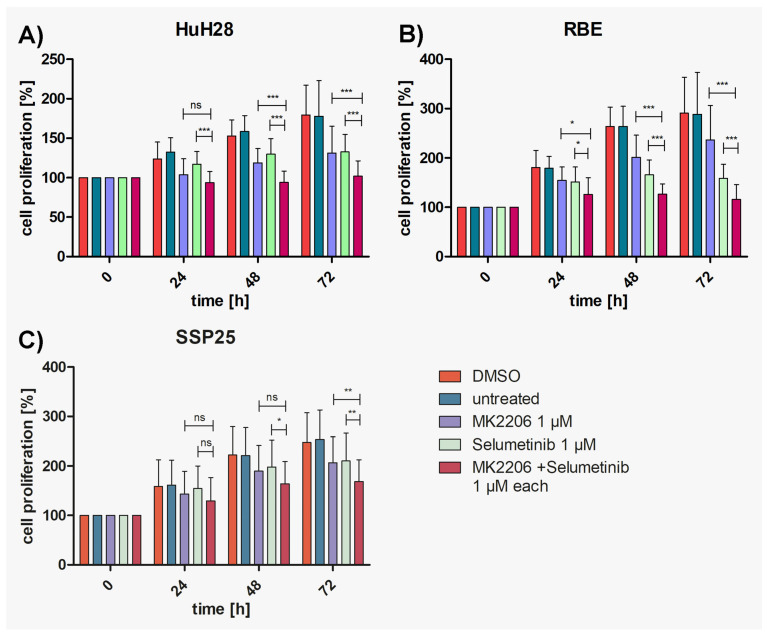
Proliferation assays of ICC cell lines with MK2206 and selumetinib, dual compared with single treatments. (**A**) HuH28; (**B**) RBE; (**C**) SSP25. Cell numbers were calculated after 0, 24, 48 and 72 h. Application of each 1 µM MK2206 and selumetinib. Mean of *n* = 3 independent experiments with eight replicates are shown, as well as standard deviation and statistical significance calculated with two-way ANOVA. Note highly significant effects of dual treatment in RBE starting after 24 h, in HuH28 starting after 48 h, and in SSP25 after 72 h. ns = not significant. * *p* ≤ 0.05, ** *p* ≤ 0.01, *** *p* ≤ 0.001.

Compared with single MK2206 (0.5 µM) treatment, dual inhibition with both 0.5 µM MK2206 and selumetinib significantly decreased the proliferation of HuH28 and RBE cells after 72 h (Figure 3A,B), with 37% growth inhibition in HuH28 and 52% in RBE (Supplementary Figure S1A,B). No additive effect was observed in SSP25 with each 0.5 µM (Figure 3C). However, the dual treatment of SSP25 with 1 µM induced a significant decrease in cell proliferation after 72 h (Figure 4C), with 32% growth inhibition (Supplementary Figure S1C).

The cell line HuH28 showed a strong significant decrease in cell number after 48 h of dual treatment with 1 µM compared with each single treatment with MK2206 or selumetinib (Figure 4A), with 39% growth inhibition (Supplementary Figure S1A).

In RBE, dual treatment with 1 µM induced a significant decrease in cell proliferation compared to the respective single treatments already after 24 h (reaching 30% growth inhibition; see: Supplementary Figure S1B), which was highly significant (*p* ≤ 0.001) thereafter (Figure 4B), with 60% growth inhibition after 72 h; see Supplementary Figure S1B. In sum, all three cell lines responded more strongly and earlier to the double treatment than to the respective single treatments. SSP25 showed the highest resistance and responded the latest.

### 3.4. MK2206 Alone or in Combination Effectively Inhibits Phosphorylation of AKT (Ser473)

**Figure 5:**
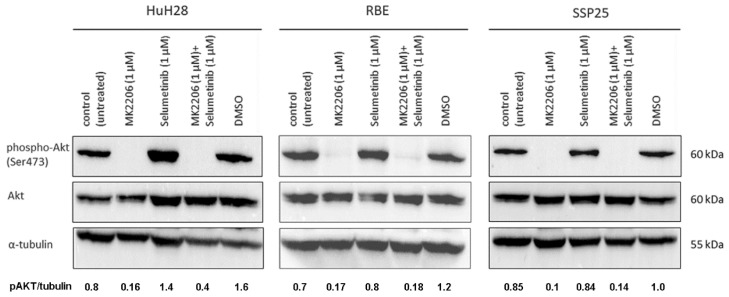
Western blot analysis of phospho-AKT (Ser473) and total AKT in ICC cell lines. A representative blot for HuH28, RBE and SSP25 of *n* = 3 independent experiments is shown. Loading control was performed against α-tubulin. Note the distinct downregulation of pAKT by MK2206 alone or in combination with selumetinib. The quantification of pAKT was normalized to tubulin (pAKT/tubulin), and the mean value of *n* = 3 experiments is shown.

In all three ICC cell lines, single inhibition with MK2206 as well as dual inhibition with MK2206 and selumetinib caused a significant downregulation of phospho (p)-AKT at Ser473 after 12 h. Total AKT protein and loading control α-tubulin remained unchanged throughout the treatment (Figure 5).

### 3.5. Selumetinib Alone or in Combination Effectively Inhibits ERK1/2 Phosphorylation (Thr202/Tyr204)

**Figure 6:**
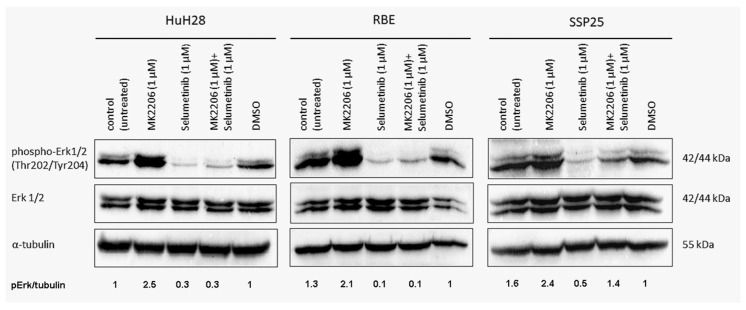
Western blot analysis with phospho-ERK1/2 in ICC cell lines. A representative blot of HuH28, RBE and SSP25 of *n* = 3 independent experiments is shown. Loading control was performed against α-tubulin. The quantification of pERK1/2 was normalized to α-tubulin (pErk/tubulin), and the mean value of *n* = 3 experiments is shown. Note the downregulation of pERK1/2 by selumetinib and upregulation by MK2206. Dual application also downregulates pERK1/2 in HuH28 and RBE, but in SSP25, the effect becomes neutralized.

In all three ICC cell lines, the signal of phospho (p)-ERK1/2 (Thr202/Tyr204) was downregulated after 12 h through selumetinib, both in single and in dual inhibition with MK2206. Interestingly, selumetinib inhibits ERK1/2 phosphorylation in SSP25, but in combination with MK2206, the inhibitory effect is neutralized when compared to the DMSO controls (Figure 6). However, our proliferation studies show an additive effect after 72 h (Figure 4C). The upregulation of pERK1/2 by single MK2206 was observed in all three cell lines, indicating interactions between the two pathways (Figure 6). The total ERK1/2 protein and α-tubulin remained unchanged. In sum, selumetinib effectively inhibits ERK1/2 phosphorylation, but AKT inhibition with MK2206 increases pERK1/2. In SSP25, this effect was even seen after dual treatment.

### 3.6. Dual Inhibition with MK2206 and Selumetinib Causes Cell Cycle Arrest in ICC Cell Lines

In order to determine if apoptosis may be responsible for the decrease in cell numbers, we used WB analysis against cleaved caspase-3 and qPCR against Apoptosis-inducing factor mitochondria-associated 1 (AIFM1).

**Figure 7:**
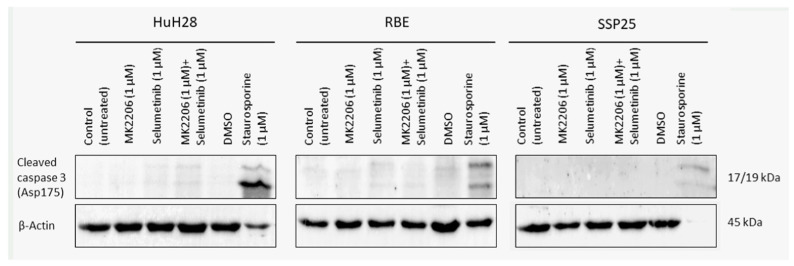
Western blot analysis of cleaved caspase-3 in ICC cell lines. A representative blot of HuH28, RBE and SSP25 of *n* = 3 independent experiments is shown. Loading control was performed against β-actin. For positive control, cells were treated with 1 µM staurosporine for 12 h, which reduced cell numbers, especially in SSP25.

**Figure 8:**
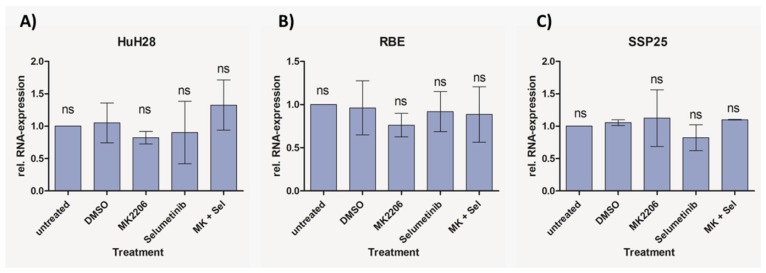
Relative mRNA expression of AIFM1 in ICC cell lines measured with qPCR. Treatment of HuH28 (**A**), RBE (**B**) and SSP25 (**C**) with each 1 µM inhibitor as indicated. Mean and standard deviation of *n* = 3 independent experiments are shown. Untreated control probe was used as reference. No regulation of AIFM1 was noted. ns = not significant.

We did not observe cleaved caspase-3 after inhibitor treatment in all three ICC cell lines (Figure 7). As a positive control, we incubated cells with 1 µM staurosporine, which induces caspase-dependent and -independent apoptosis [30]. Especially in SSP25, staurosporine was highly effective and dramatically reduced cell numbers. As a result, only very weak signals could be found after 12 h staurosporine incubation (Figure 7). The transcriptional regulation of AIFM1 during apoptosis has been described [31,32]. Our qPCR studies did not show any AIFM1 regulation by the two inhibitors (Figure 8), which again excludes the involvement of apoptosis as a contributor to cell number reduction in the experiments.

**Figure 9:**
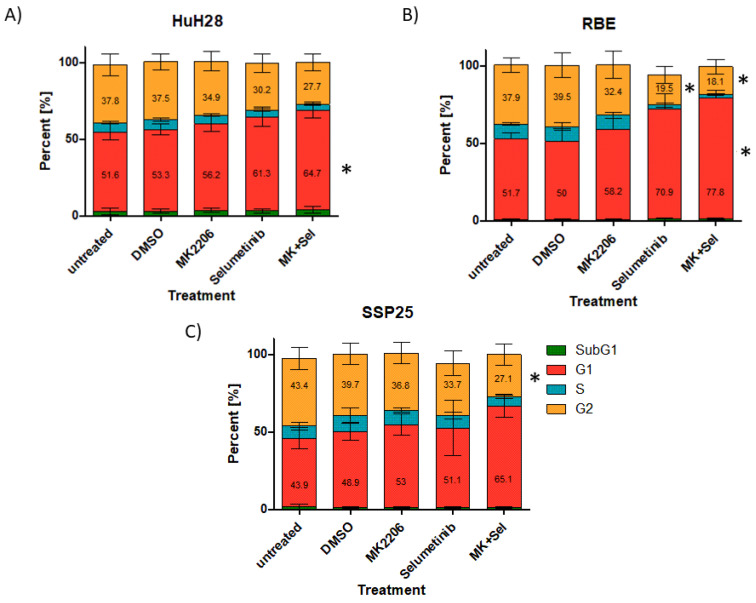
Flow cytometry analysis of ICC cell lines. Treatment of HuH28 (**A**), RBE (**B**) and SSP25 (**C**) with each 1 µM inhibitor as indicated. Mean and standard deviation of percentage of each cell cycle phase of *n* = 5 independent experiments are shown. Significance was calculated in comparison to DMSO controls with one-way ANOVA and * *p* < 0.05. Note significant effects of dual inhibitor treatment. Number of dying cells in sub-G1 phase is consistently very low (0.6–2.1%) and not influenced by the inhibitors.

Flow cytometry showed a slight increase in cells in the G1 phase after a single treatment of HuH28 with 1 µM MK2206 or selumetinib. The percentage of cells in the G2 phase slightly decreased. Dual inhibition induced a significant increase in cells in the G1 phase as compared to the DMSO control (Figure 9A). Similar results were obtained in RBE cells. Here, selumetinib induced a significant decrease in cells in G2, while dual inhibition caused both a significant decrease in the G2 phase and a significant increase in G1-phase cells (Figure 9B). In SSP25, we observed a tendency for an increase in G1 and a significant decrease in cells in the G2 phase after dual inhibition (Figure 9C). For all three cell lines, we made sure to include dying cells in the supernatant. In all experiments and controls, this population is consistently extremely low, and there are no inhibitor effects (1.5–1.8% in DMSO controls vs. 0.6–2.1% in experimental groups). In sum, we did not notice any changes in the sub-G0/G1 phase (Figure 9), which is characteristic for apoptotic and fragmented cells [33]. Typical FACS histograms are shown in Supplementary Figure S3.

## 4. Discussion

### 4.1. Dual Inhibition of MAPK/ERK and PI3K/AKT/mTOR Is Highly Effective in ICC

Because of the increasing incidence of CCA and the limited therapeutic options, new drugs are urgently needed. A more specific combination of targeted drugs could be a solution to the problem. It has become increasingly clear that from both the molecular and surgical point of view, CCA is not a uniform tumor entity. Topographically, CCA is subdivided into intrahepatic (ICC) and extrahepatic (ECC) tumors, and ECC can be further divided into perihilar and distal types [4,5,6]. Based, most likely, on the differential embryonic origin of intra- and extrahepatic cholangiocytes [17,18], distinct molecular differences between ICC and ECC have been observed. ICC patients are more likely to have *FGFR2* fusions, *FGFR* mutations and *IDH1* mutations than ECC patients. ECC patients, however, more commonly show *KRAS*, *TP53*, *SMAD4* and *APC* mutations [16]. A transcriptomic analysis of ICC and ECC cells identified subgroups which revealed different tumor biology. Thereby, gene expression profiles from 340 ICC and 203 ECC patients were compared. ICC cells were enriched in molecular pathways such as EMT, IL6, RTK-RAS-PIK3K and DNA repair. ECC cells were enriched in EGFR, VEGF signaling and integrin pathways [31,34]. This suggests that differential treatment for the subtypes of CCA will be necessary to achieve promising therapeutic results.

Here, we focused on ICC and studied three human ICC cell lines in vitro (HuH28, RBE and SSP25) in order to determine the effects of inhibitors of two common tumor signaling pathways. We tested the AKT inhibitor MK2206 and the MEK inhibitor selumetinib (AZD6244) and studied proliferation, the phosphorylation of signaling molecules of the PI3K/AKT/mTOR and the MAPK/ERK pathways and the cell cycle. We used concentrations of MK2206 well below the IC50 values, assuming that additive or synergistic effects should be seen at a significantly reduced dose. Basically, this type of research is not new. However, it has so far been performed on uncharacterized CCA cell lines and on ECC lines [15]. We show that ICC cell lines behave differently in their response to the inhibitors. Thereby, single treatment with MK2206 or selumetinib induced the inhibition of proliferation, and the effect was clearly enhanced by the dual application of the inhibitors (see Figure 3 and Figure 4). Thereby, HuH28 and RBE were more sensitive, but at a 1 µM dosage of inhibitors, SSP25 responded significantly, too. Western blot analyses with antibodies against p-AKT (Ser473) and p-ERK confirmed the effectiveness of the two inhibitors. Thereby, single as well as dual inhibition showed the downregulation of the phosphorylation of the respective proteins in all three ICC cell lines (see Figure 5 and Figure 6). A potential direct interaction between the two pathways was well visible. The WB pERK quantification showed that selumetinib significantly downregulates pERK, while MK2206 upregulates pERK both alone and, in SSP25, also in combination with selumetinib (see Figure 6). We have previously observed the upregulation of pERK by MK2206 in various liver cancer cell lines [29]. Such interactions have been observed in other tumor types, including melanoma, colorectal, pancreatic and breast carcinoma [35,36], and were also reported for CCA [15]. For a review on the crosstalk of the pathways, see [37,38].

### 4.2. Dual Inhibition of MAPK/ERK and PI3K/AKT/mTOR Does Not Induce Apoptosis in ICC

In contrast to previous studies on CCA cell lines [15,39], we did not observe any signs for apoptosis or other forms of cell death by the dual inhibition of the MAPK/ERK and PI3K/AKT/mTOR signaling pathways in ICC. There was no expression of cleaved caspase-3, which is the dominant effector caspase [40,41,42], or expression of AIFM1 (see Figure 7 and Figure 8). In previous studies, antibodies against pro-caspase-3 and -9 were used to identify apoptosis [39]. The number of dying cells in the sub-G0/G1 phase was neglectable and only about 1%, as in the controls (see Figure 9). Neither single treatment with MK2206 or selumetinib nor dual treatment with both inhibitors reduced cell numbers below baseline. The inhibitors effectively induced a proliferation block but not apoptosis or other types of cell death. In line with this, we observed cell cycle arrest, which was evident from a significant increase in cells in the G1 phase (see Figure 9). Since autophagy is inhibited by mitosis [43], prolonged cell cycle arrest might increase the susceptibility of ICC to selective autophagy induction, as an additional therapeutic target [44].

The in vitro results seem to suggest that the dual application of MK2206 and selumetinib in ICC may stop tumor growth but may probably not eliminate residual tumors. A phase I dose escalation study of MK2206 and selumetinib in therapy-refractory solid tumors has shown clinical effects in patients with different tumors with *K-RAS* mutations. However, the study also shows that not all patients responded well, and it was assumed that this was due to the heterogeneity of tumor biology. However, the study did not include patients with biliary or hepatic carcinoma [45]. A biomarker-driven phase II trial in patients with colorectal cancer has also been conducted for MK2206 and selumetinib, in which no clinical efficacy was observed [46]. Whether the dual inhibition with MK2206 and selumetinib could be a treatment option for patients with ICC must be critically scrutinized and tested. Proliferation block may even induce resistance against standard therapies, which target highly proliferative cells. However, the inhibition of important signaling pathways, including PI3K-AKT-mTOR, not only affects tumor cells but also the tumor microenvironment and immune cells. Anti-tumorigenic macrophage polarization and increased antigen presentation on dendritic cells have been observed in various tumor types [47,48].

## Figures and Tables

**Table 1:** Primary antibodies and manufacturers.

Primary Antibody	Manufacturer
β-actin—HRP conjugated	CellSignaling (Cambridge, UK)
phospho-AKT (Ser473)	CellSignaling (Cambridge, UK)
AKT	CellSignaling (Cambridge, UK)
anti-MLKL (Phospho S358) [ERP9514]	Abcam (Cambridge, UK)
anti-MLKL [ERP17514]	Abcam (Cambridge, UK)
cleaved caspase-3 (Asp175)	CellSignaling (Cambridge, UK)
phospho-p44/42 MAPK (ERK1/2) (Thr202/Tyr204)	CellSignaling (Cambridge, UK)
p44/42 MAPK (ERK1/2)	CellSignaling (Cambridge, UK)
α-tubulin—HRP conjugated	CellSignaling (Cambridge, UK)

## Data Availability

All data are included in the manuscript and the Supplementary Material data files. Further inquiries can be directed to the corresponding author.

## Supplementary Materials

The following supporting information can be downloaded at https://www.mdpi.com/article/10.3390/cimb46070439/s1, Figure S1: Percentage of inhibition of ICC cell lines by selumetinib, MK2206 and dual treatment. (A) HuH28. (B) RBE. (C) SSP25. Graphs show percentage of inhibition of different concentrations of inhibitors at 24 h, 48 h and 72 h. Note more effective inhibition of dual treatment as compared to single treatments with corresponding dosages. Figure S2: Proliferation assays of ICC cell lines with a combination of MK2206 and selumetinib. (A) HuH28; (B) RBE; (C) SSP25. Cell numbers were calculated after 0, 24, 48 and 72 h, after application of MK2206 and selumetinib of each 0.5 µM and 1 µM. Medium and DMSO controls are shown. Statistical analyses were performed compared to DMSO controls. Mean of *n* = 3 independent experiments with eight replicates are shown, as well as standard deviation and statistical significance. Note highly significant effects in HuH28 and RBE after 24 h and in SSP25 starting after 48 h. ns = not significant. Figure S3: Typical histograms of FACS analyses of ICC cell lines treated for 24 h with each 1 µM selumetinib, MK2206 or dual application. (A) HuH28. (B) RBE. (C) SSP25. Based on intensity of PI staining, phases of cell cycle were visualized (G1, S, G2 and sub-G1). Representative histogram was chosen out of *n* = 5 individual experiments.

## Abbreviations

APC	Adenomatosis Polyposis Coli
AIFM1	Apoptosis-inducing factor mitochondria-associated 1
AKT	Protein kinase B
CCA	Cholangiocellular carcinoma
DMSO	Dimethyl sulfoxide
ECC	Extrahepatic cholangiocellular carcinoma
EMT	Epithelial–mesenchymal transition
ERK	Extracellular signal-regulated kinase
FGFR2	Fibroblast growth factor receptor 2
ICC	Intrahepatic cholangiocellular carcinoma
IDH1	Isocitrate dehydrogenase-1
KRAS	*K*irsten *Ra*t *S*arcoma Viral Oncogene Homolog
MAPK	Mitogen-activated protein kinase
MEK	Mitogen-activated protein kinase kinase
MLKL	Mixed Lineage Kinase Domain Like
mTOR	Mammalian target of rapamycin
PBS	Phosphate-buffered saline
PI3K	Phosphatidylinositol-3-kinase
SMAD4	Smad family member 4
TP53	Tumor protein P53
