# Supplementary material for: Dual Inhibition of PI3 Kinase and MAP Kinase Signaling Pathways in Intrahepatic Cholangiocellular Carcinoma Cell Lines Leads to Proliferation Arrest but Not Apoptosis
Source: Curr Issues Mol Biol. 2024 Jul 13;46(7):7395–410. doi: 10.3390/cimb46070439 (PMC11276521; doi:10.3390/cimb46070439)
Supplement: Supplementary file 1 [file cimb-46-00439-s001.zip › cimb-3072908-supplementary.pdf]

C

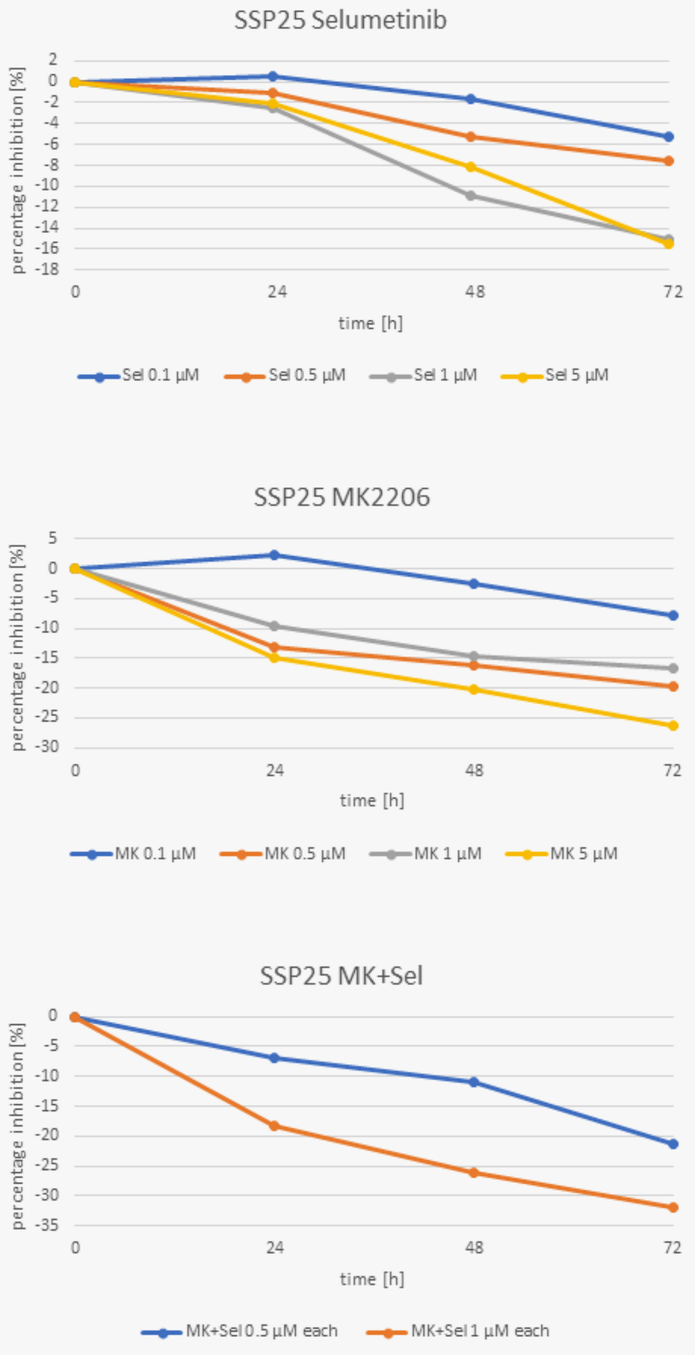
SSP25 Selumetinib
percentage inhibition [%]
2
0
-2
-4
-6
-8
-10
-12
-14
-16
-18
0
24
48
72
time [h]
Sel 0.1 µM
Sel 0.5 µM
Sel 1 µM
Sel 5 µM
SSP25 MK2206
percentage inhibition [%]
5
0
-5
-10
-15
-20
-25
-30
0
24
48
72
time [h]
MK 0.1 µM
MK 0.5 µM
MK 1 µM
MK 5 µM
SSP25 MK+Sel
percentage inhibition [%]
0
-5
-10
-15
-20
-25
-30
-35
0
24
48
72
time [h]
MK+Sel 0.5 µM each
MK+Sel 1 µM each

B

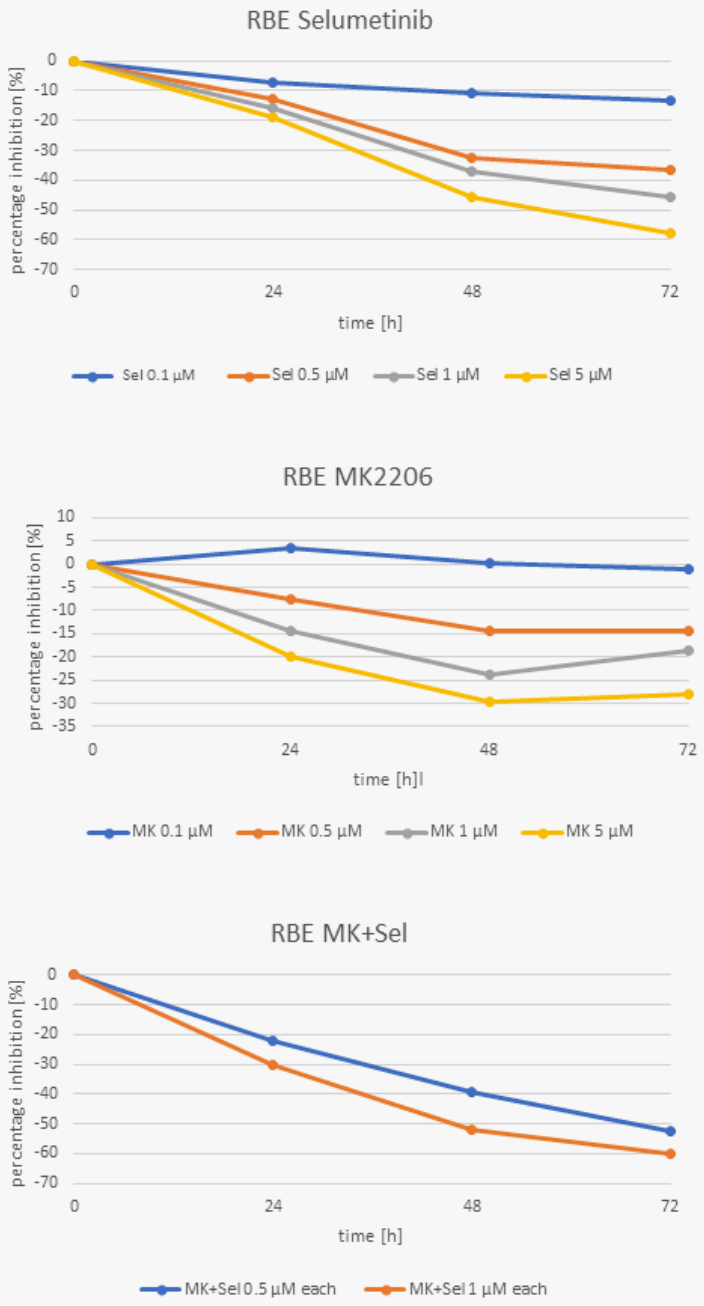
RBE Selumetinib
percentage inhibition [%]
0
-10
-20
-30
-40
-50
-60
-70
0
24
48
72
time [h]
Sel 0.1 µM
Sel 0.5 µM
Sel 1 µM
Sel 5 µM
RBE MK2206
percentage inhibition [%]
10
5
0
-5
-10
-15
-20
-25
-30
-35
0
24
48
72
time [h]l
MK 0.1 µM
MK 0.5 µM
MK 1 µM
MK 5 µM
RBE MK+Sel
percentage inhibition [%]
0
-10
-20
-30
-40
-50
-60
-70
0
24
48
72
time [h]
MK+Sel 0.5 µM each
MK+Sel 1 µM each

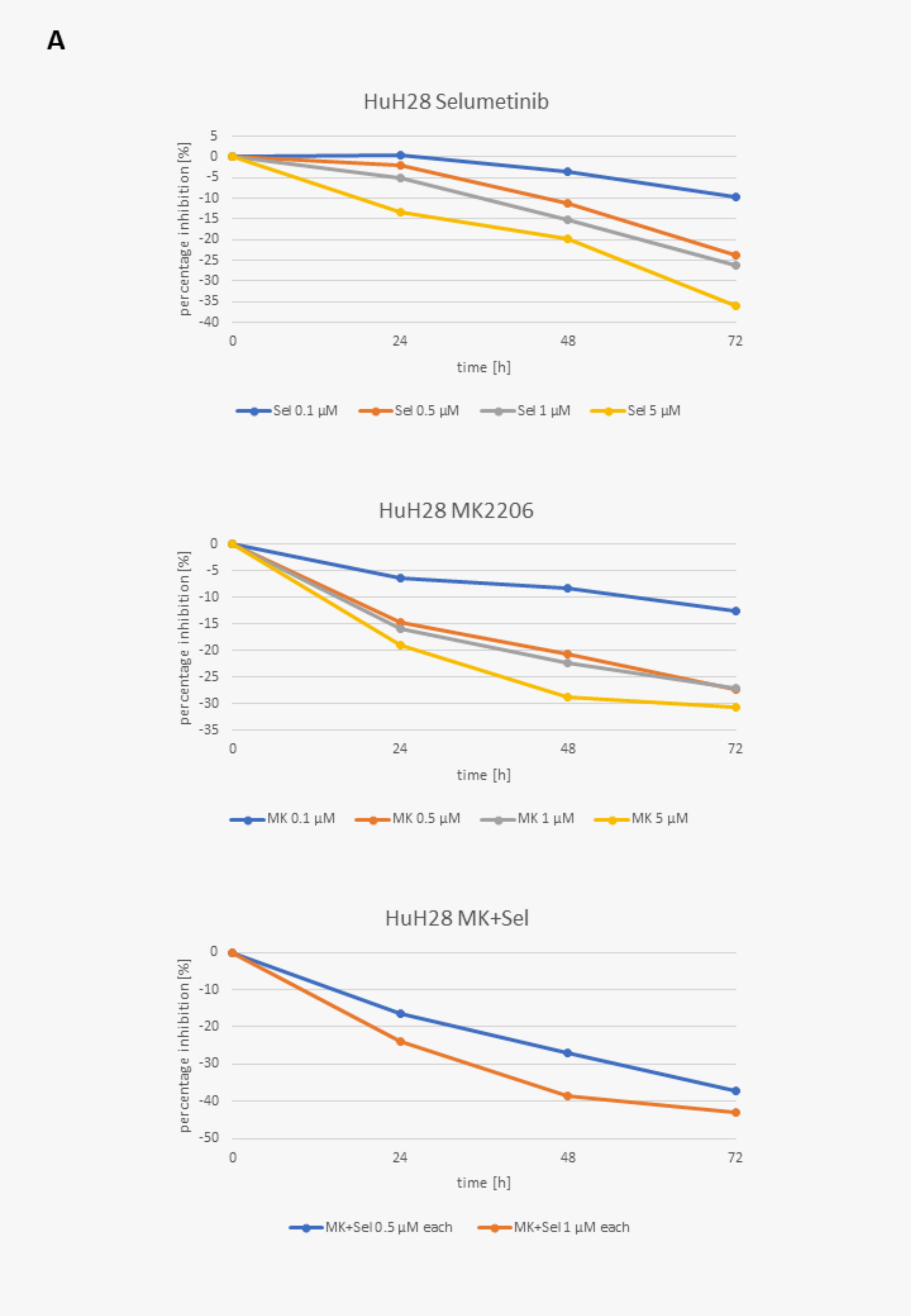

Suppl. Figure S1: Percentage of inhibition of ICC cell lines by selumetinib, MK2206 and dual treatment. A) HuH28. **B)** RBE. **C)** SSP25. Graphs show the percentage of inhibition of different concentrations of inhibitors at 24h, 48h and 72h. Note the more effective inhibition of dual treatment as compared to the single treatments with corresponding dosages.

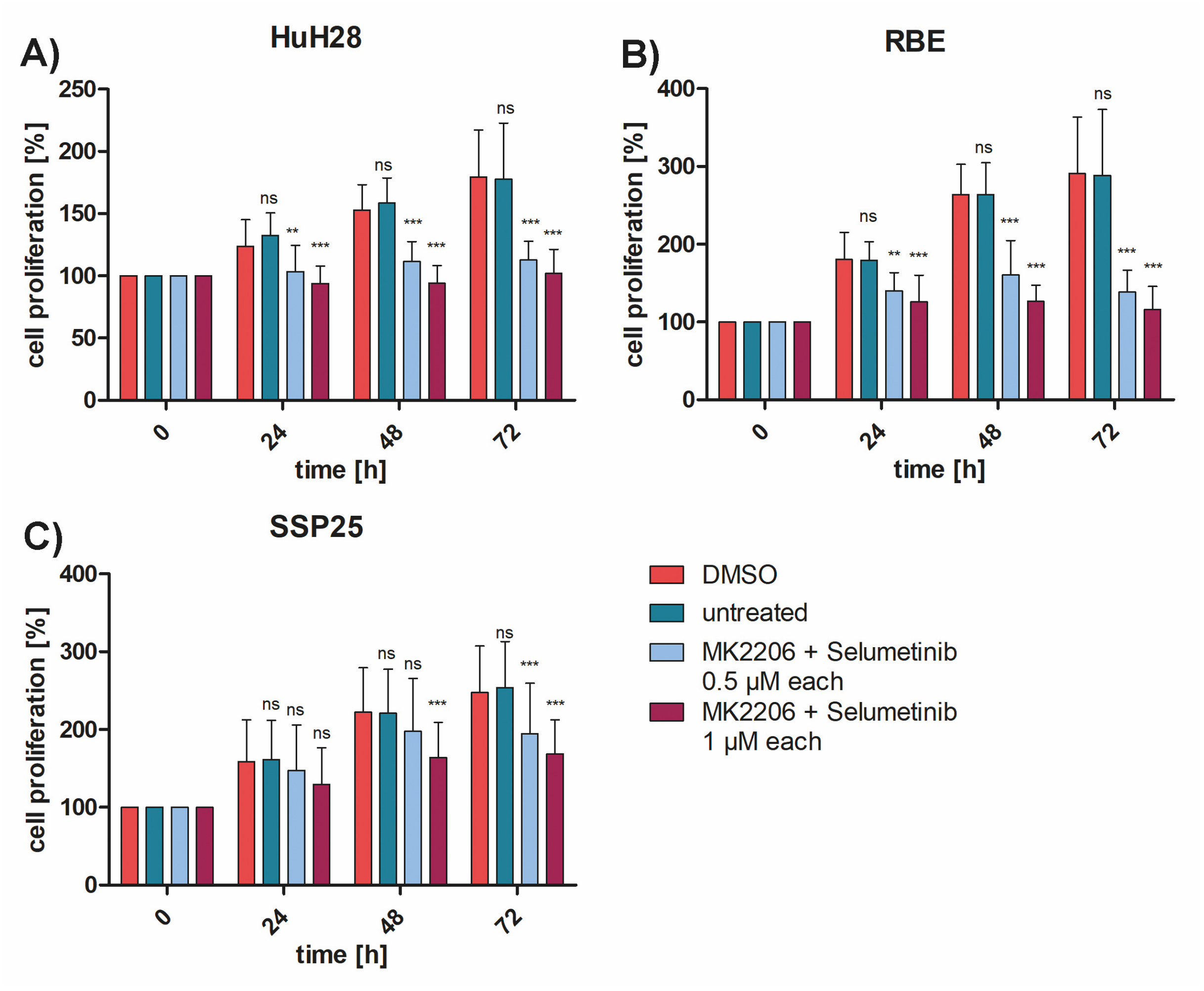

Suppl. Figure S2: Proliferation assays of ICC cell lines with a combination of MK2206 and selumetinib. A) HuH28; **B**) RBE; **C**) SSP25. Cell numbers were calculated after 0, 24, 48 and 72 hours (h), after application of MK2206 and selumetinib of each 0.5 µM and 1 µM. Medium and DMSO controls are shown. Statistical analyses were performed compared to DMSO controls. Mean of n=3 independent experiments with each eight replicates are shown, as well as standard deviation and statistical significance. Note highly significant effects in HuH28 and RBE after 24h, and in SSP25 starting after 48h. ns=not significant.

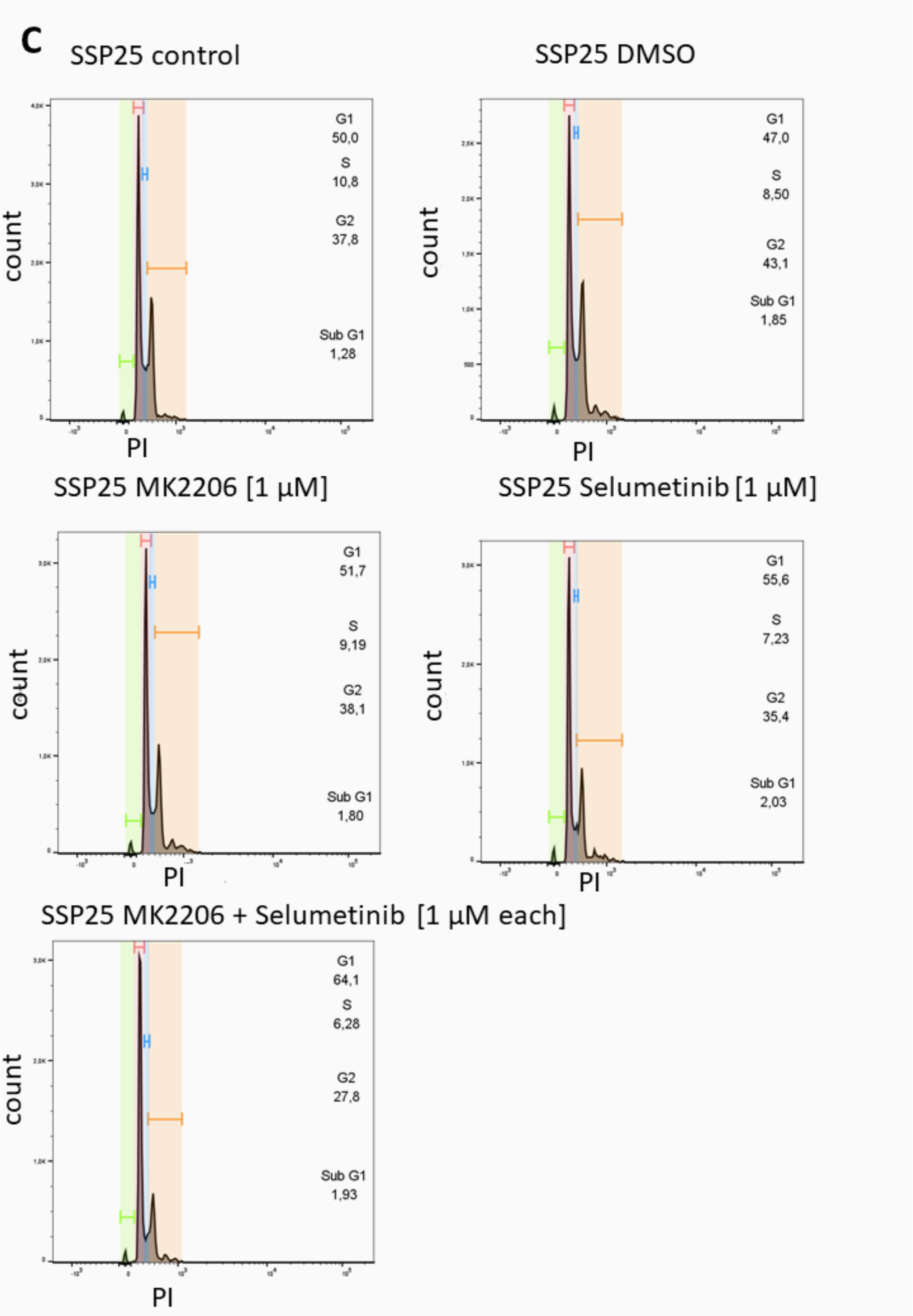
c
SSP25 control
G1
50,0
S
10,8
G2
37,8
Sub G1
1,28
count
PI
SSP25 DMSO
G1
47,0
S
8,50
G2
43,1
Sub G1
1,85
count
PI
SSP25 MK2206 [1 µM]
G1
51,7
S
9,19
G2
38,1
Sub G1
1,80
count
PI
SSP25 Selumetinib [1 µM]
G1
55,6
S
7,23
G2
35,4
Sub G1
2,03
count
PI
SSP25 MK2206 + Selumetinib [1 µM each]
G1
64,1
S
6,28
G2
27,8
Sub G1
1,93
count
PI

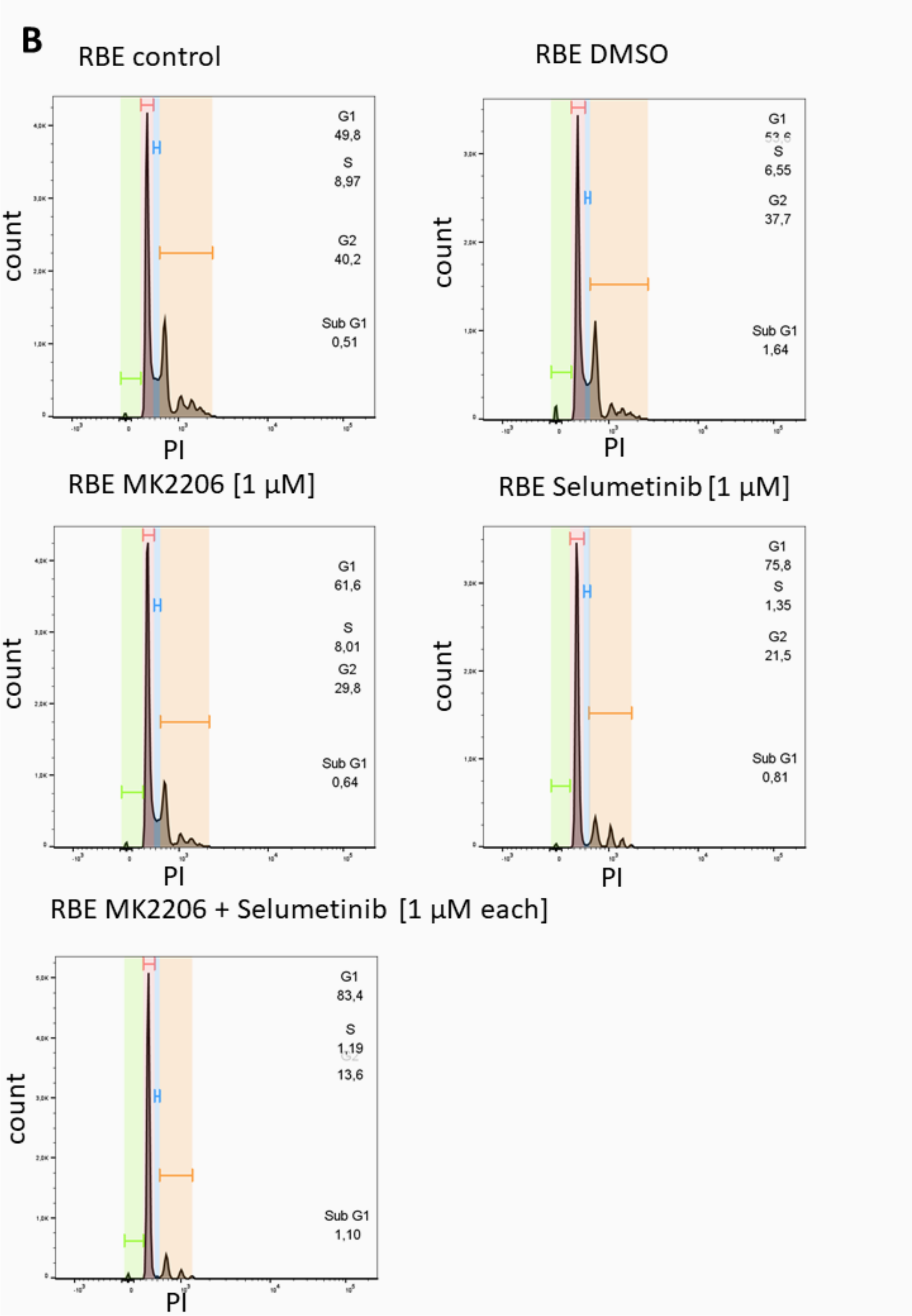
B
RBE control
G1
49,8
S
8,97
G2
40,2
Sub G1
0,51
count
PI
RBE DMSO
G1
S
6,55
G2
37,7
Sub G1
1,64
count
PI
RBE MK2206 [1 µM]
G1
61,6
S
8,01
G2
29,8
Sub G1
0,64
count
PI
RBE Selumetinib [1 µM]
G1
75,8
S
1,35
G2
21,5
Sub G1
0,81
count
PI
RBE MK2206 + Selumetinib [1 µM each]
G1
83,4
S
1,19
13,6
Sub G1
1,10
count
PI

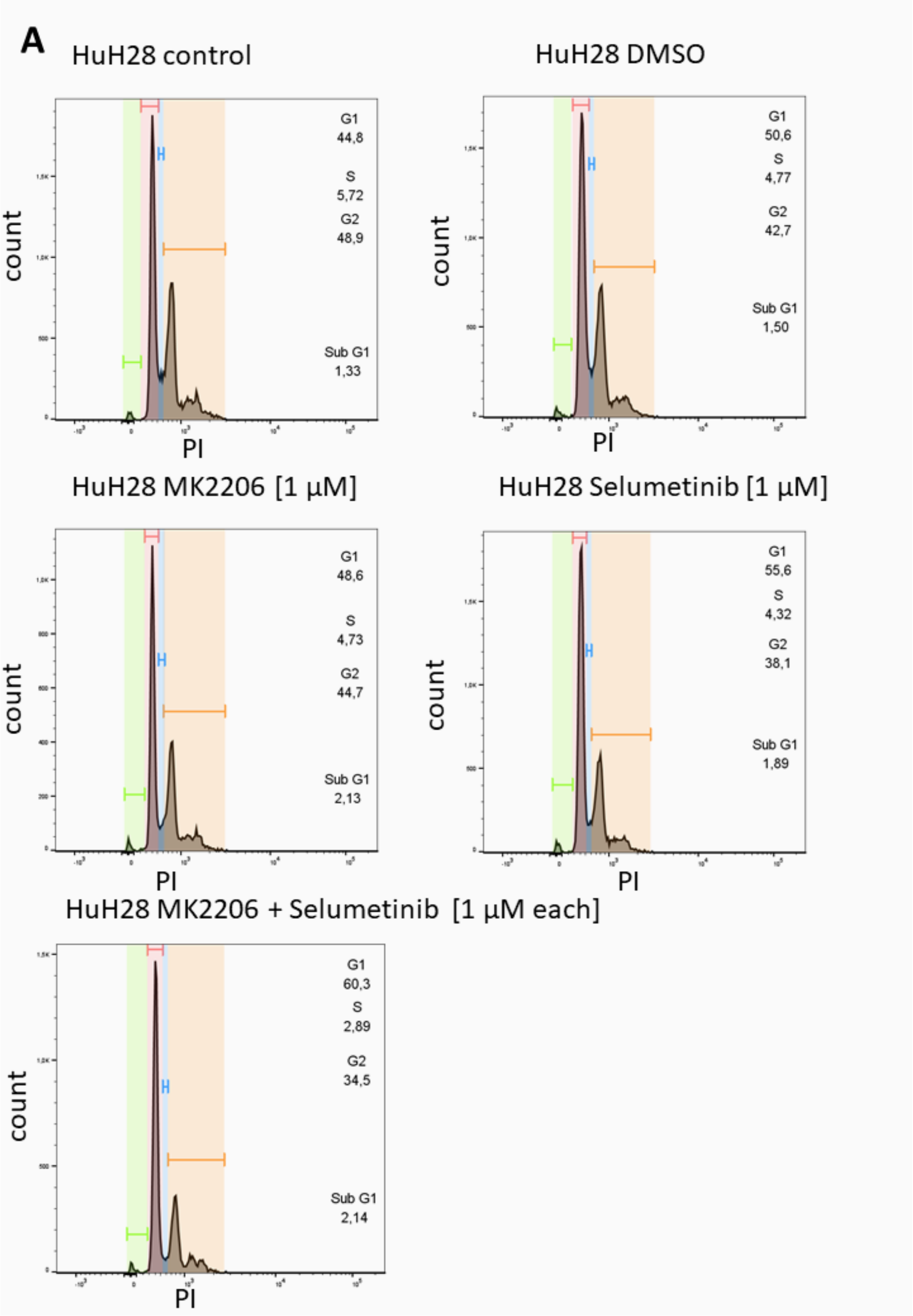

Suppl. Figure S3: Typical histograms of FACS analyses of ICC cell lines treated for 24 h with each 1µM selumetinib, MK2206 or dual application. A) HuH28. **B**) RBE. **C)** SSP25. Based on the intensity of the PI staining, phases of the cell cycle were visualized (G1, S, G2 and subG1). A representative histogram was chosen out of n=5 individual experiments.
